# Effect of Dietary Exposure to Low-Density Polyethylene Microplastics and Their Potential Role as Estrogen Vectors In Vivo

**DOI:** 10.3390/cimb47090701

**Published:** 2025-08-30

**Authors:** Noura Al-Jandal, Azad Ismail Saheb, Abdulaziz Alkhubaizi, Abrar Akbar, Enas Al-Hasan, Sumaiah Hussain, Hamad Al-Mansour

**Affiliations:** Environmental Pollution and Climate Program, Environment and Life Sciences Research Centre, Kuwait Institute for Scientific Research, P.O. Box 24885, Safat, Kuwait City 13109, Kuwait

**Keywords:** microplastics, low-density polyethylene, estrogens, marine ecosystems

## Abstract

Microplastics (MPs) are a growing environmental concern due to their ability to adsorb hazardous chemicals, such as estrogens, and be ingested by marine organisms. This study focuses on low-density polyethylene (LDPE), a polymer widely used in Kuwait, to assess its role as a carrier of endocrine-disrupting chemicals (EDCs), specifically estrogens. Biological effects were evaluated using biomarkers such as cytochrome P450 1A (CYP1A) and vitellogenin (Vtg) gene expression. Virgin LDPE MPs were exposed to influent and effluent from a wastewater treatment plant (WWTP) for four weeks to facilitate estrogen absorption. The MPs were then incorporated into fish feed pellets for dietary exposure experiments. Fish were divided into three treatment groups—exposed to either virgin MPs, WWTP-influent MPs, or WWTP-effluent MPs—and monitored over four weeks. The results showed that WWTP-exposed MPs carried detectable levels of estrogen, leading to physiological effects on yellowfin bream. Fish in the control group, which received MP-enriched diets without estrogen, experienced significant weight loss due to nutrient deprivation. In contrast, weight patterns in the treatment groups were influenced by estrogen exposure. The condition factor (CF) decreased across groups during the experiment but remained within acceptable health ranges. A significant reduction in the hepatosomatic index (HSI) was observed in the effluent-exposed group, likely due to lower estrogen levels reducing physiological stress. The findings confirm that LDPE MPs can act as carriers for estrogens, impairing fish growth and metabolism while disrupting biological processes such as cytochrome oxidase function. These results highlight the potential risks of MPs in marine ecosystems and underscore the need for further research to understand their long-term effects.

## 1. Introduction

The improper management and disposal of plastic waste contributes to many environmental pollution problems [1,2]. Microplastics (MPs) are formed when plastic waste is introduced to the environment and slowly breaks down. Plastics that are smaller than 5 mm are usually classified as MPs [1,2,3]. The widespread use of MPs has made them a primary environmental concern in recent years [4,5]. Kuwait is the second-highest producer of solid plastic waste in the Arabian Gulf Cooperation Council (GCC), generating approximately 18.2% of the total. Low-density polyethylene (LDPE) polymers are among the most common polymer types used in Kuwait [6].

Environmental pollutants are transported by MPs, which are normally fragmented particles ranging between 0.1 and 5 mm in size [7]. MPs are primarily ingested [8], inhaled [9], and absorbed from the water. Exposure to plastic particles and their associated chemicals normally occurs during day-to-day activities [9].

Combined with fragmentation and strong hydrophobicity, MPs have a high surface area-to-volume ratio, enhancing chemical sorption (adsorption and absorption). According to their size, shape, chemical composition, surface charge, and hydrophobicity, MPs can be categorized based on their toxicity [9,10,11,12]. Chemicals are transferred from fluids (liquids and gases) to solids by sorption [12,13]. Under artificial laboratory conditions, MPs readily accumulate waterborne persistent bioaccumulated toxic substances (PBTs) [14,15,16]. Hydrophobic organic contaminants (HOCs) prefer nonpolar surfaces due to their surface polarity [17]. MPs have been proposed to deliver environmental contaminants and transport contaminants between environments [18,19], but their environmental relevance remains unclear.

While MPs contain a mixture of chemicals added during manufacturing, they also absorb contaminants from their surroundings [15], including polychlorinated biphenyls (PCBs), polycyclic aromatic hydrocarbons (PAHs), chlorinated pesticides, and trace metals [18,20,21]. In aquatic and terrestrial environments, MPs interact with hydrophobic organic compounds (HOCs) through sorption–desorption [2]. The intake of MPs has been reported [22,23] as an alternative route to exposure to HOCs [24]. Due to their small size, hydrophobicity, high stability, and mobility, MPs show strong sorption and enrichment characteristics towards HOCs [25].

According to several studies, MPs have a minor effect on the influx of chemicals into biota. Chemical exposure from MPs is insignificant compared to exposure from food and other particulates [26,27,28,29,30]. The amount of particulates in aquatic environments far exceeds current levels. By 2030, plastic emissions are expected to reach 53 million metric tons [31].

The ability of different polymers to absorb contaminants and then transfer them to aquatic flora and fauna is quite restricted. MPs also contain additives of polymeric raw material, and the sorption of environmental pollutants on MPs has raised concerns [7,32]. There are two types of plastic polymers, glassy (adsorbent) and rubbery (absorptive). Amorphous regions in rubbery polymers, such as polyethylene (PE), allow chemical sorption into the plastic (absorption). On the other hand, glassy polymers have more condensed and crosslinked chain structures, such as polystyrene (PS) and polyethylene terephthalate (PET), which can adsorb HOCs to their surfaces or chemically partition them into nanosized pores [24,33,34]. Additionally, chemical hydrophobicity has been identified as one of the significant properties governing chemical sorption onto polymer particles, which plays a critical role in chemical sorption and desorption, determining the extent of biota’s chemical absorption. The chemical hydrophobicity of polymer particles has also been identified as a significant property governing chemical sorption onto polymer particles, determining biota’s chemical absorption extent. As a result of their composition, size, and ability to adsorb, release, and partition toxic and endocrine-disrupting chemicals (EDCs), they pose a significant threat to marine ecosystems [18,35].

Ingested particles pass through the stomach and intestine and are excreted or absorbed by the intestinal epithelium. MPs may disrupt cell membranes, trigger oxidative stress, and cause imbalances in the gut microbiota and inflammation [36,37]. In a recent study, artificial intestinal fluid enhanced chemical desorption from MPs, resulting in intestinal absorption and distribution [38].

Marine species, including economically significant species like gilthead seabream (*Sparus aurata*) Linnaeus 1758, may suffer physical effects due to MP ingestion such as satiation and abrasion depending on the ingested particles [39,40], physiological effects like weight loss and the condition index [41], and ecotoxicological effects due to the ingestion of MPs with the bioaccumulation of chemicals [40,42,43]. Laboratory and field studies have documented aquatic organisms ingesting MPs, including fisheries and aquaculture species of commercial importance [20,44]. Fish may intentionally consume MPs by mistaking them for natural prey or when the plastic is already in the body of the wild prey [45,46]. There is an increasing concern of multiple effects of MPs on fish due to their ability to harm them by clogging digestive tracts, changing lipid metabolism, cytotoxicity, and fish behavior [47], and causing intestinal damage, weight loss, liver necrosis, and even fish death when consumed [48]. Polyethylene MPs in the marine environment cause inflammation, stress-related changes, and behavioral problems in fish when ingested [49].

Marine organisms have not been extensively exposed to MPs as a vector of HOCs through laboratory dietary exposures comparing MPs to water, suspended organic particulates, natural diets, and prey items, all of which have partition coefficients similar to plastic in the environment [17,50].

For this study, LDPE MPs were used, as they are the most used polymer material in Kuwait. The purpose of this study is to investigate the potential risk of LDPE MPs, serving as carriers of one of the EDCs, and estrogens, as well as to assess estrogens’ affinity for desorbing from LDPE MPs. Cytochrome P450 1A (CYP 1A1) and vitellogenin (Vtg) gene expression were studied as biological biomarkers of estrogen presence. It is essential to understand and predict the potential consequences of the anticipated increase in MPs in marine environments and their potential to act as vector contaminants to direct further research and monitoring in this area. We hypothesize that low-density polyethylene (LDPE) MPs can adsorb environmental estrogens and act as in vivo vectors, influencing endocrine function in fish through dietary exposure.

## 2. Materials and Methods

### 2.1. Microplastic Pellets and Fish Selection

The selected MP for this exposure is LDPE, which is an opaque, milky-white, wax-like material [51]. The production of polyethylene resin has reached 100 million tons per year, with 80 million tons of plastic waste generated annually from PE alone [52].

LDPE microplastic virgin pellets approximately 3 mm in diameter (Grand Polymer Co., Ltd., Tokyo, Japan) were purchased from the local supplier Plastic Industries Company in Kuwait. The MPs obtained were used in their original form and selected to be examined rather than secondary MPs. Only glassware or stainless-steel tools were used during all stages of sample handling and extraction. All glassware was triple-rinsed with methanol, dichloromethane, and distilled hexane to eliminate potential plastic residue. All sample processing was conducted under a laminar flow hood, which was cleaned before and after use to reduce airborne MP contamination.

The selected fish species, yellow-finned seabream (*Acanthopagrus latus*), is one of Kuwait’s most commercially and economically viable marine fish [53]. As a result of the possible estrogen exposure, the maturation stage of the fish is critical to testing the validity of our hypothesis. The examined fish were cultured and reared in our facilities to ensure the fish had not been exposed to contaminants previously.

For the dietary experiment, cultured fish were obtained from the Kuwait Institute for Scientific Research (KISR) aquaculture program. All male juvenile fish (60–110 g) were stocked and acclimatized to the laboratory’s exposure tanks before dietary exposure. The experiments were conducted in the dedicated aquaculture facilities of the KISR. Guidelines laid out by the KISR for experimentation on fish were followed. Fish sacrifice for analysis was performed using organic anesthesia to minimize fish suffering during the sacrifice. Weight and length measurements were conducted while the fish were anesthetized.

### 2.2. LDPE Microplastic Exposure in Wastewater Treatment Plant

**Figure 1:**
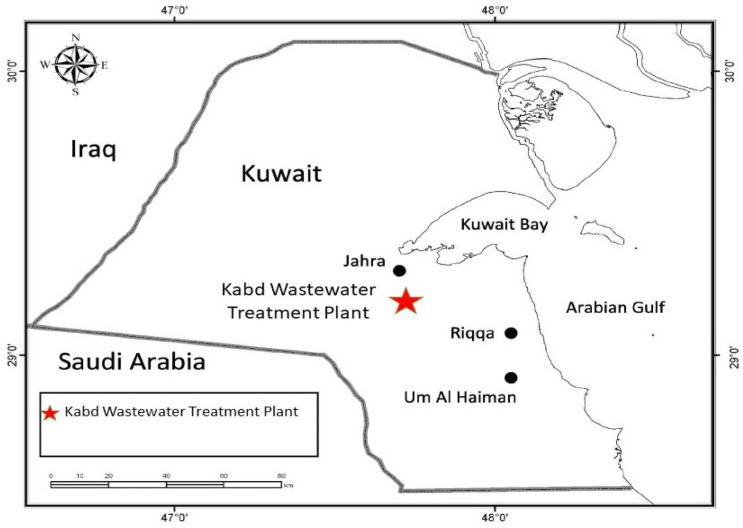
Approximate location of Kabd wastewater treatment plant.

Virgin microplastic pellets were prepared to be exposed in the Kabd wastewater treatment plant (WWTP) located in the southwest of Kuwait City (Figure 1). This plant was selected because we had previously reported that effluents in this plant were estrogenic to fish, where estrogen concentration in the influent streams ranged from 0.0 to 474 ng/L, while in the effluent streams, it was between 0.0 and 233 ng/L. In addition, the average removal rates of total estrogens were found to be 13% [54,55,56,57]. The MPs, sufficient in quantity for exposure studies, were exposed to the inflow (influent) and outflow (effluent) water for four weeks. LDPE MPs were kept in a mesh sac and transferred to the WWTP for exposure. The exposure of MPs (600 g) in the WWTP was for a 4-week duration.

By using metal mesh sacs, MP exposure in WWTP streams was tested for validity and ease of exposure.

### 2.3. Chemicals Extraction of LDPE Microplastics (Pristine and Exposed)

Virgin MPs (control) and exposed ones were extracted and analyzed for estrogen. MPs were stored in glass bottles at −30 °C in the laboratory. Before chemical extraction, MPs were air-dried for three days. To remove organic contamination, all glassware was rinsed with methanol, dichloromethane (DCM), and distilled hexane several times. Dried MPs were weighed before extraction by ultrasonication with methanol. Methanol was evaporated under nitrogen, and the extract was transferred into thick-walled V-shaped vials and evaporated to dryness under nitrogen. Estrogens were derived using the BSTFA/TMCS sialylation reagent. The vials were heated to 70 °C on a hot plate for one hour. After cooling to room temperature, the contents were dried under nitrogen. The residue was taken up in 1 mL of hexane and transferred to an autosampler vial. Samples were analyzed by gas chromatography–mass spectrometry (GC/MS) in selected ion monitoring mode (SIM).

### 2.4. Dietary Exposure of LPDE Microplastics to Fish

Both virgin and WWTP-exposed MPs were prepared for dietary exposure. The MPs were mixed with fish food and prepared as food pellets. According to previous studies, the daily intake of MPs in marine environments cannot exceed 0.3% [58]. Hence, the MP concentration in the consumed diet was calculated to be 0.15–0.3% of the required intake within a realistic limit. Semi-static tanks were prepared in triplicate for each treatment (control, influent-exposed MP-enriched diet, and effluent-exposed MP-enriched diet). Fish were randomly divided into three tanks (24 fish/treatment). The fish were allowed to acclimatize to the new conditions before the experiment began. Feeding regimes were calculated based on fish weight (2% of total body weight per day). Morning feed pellets incorporating MPs were given to the fish, and evening feed consisted of regular fish food. Water was changed in each tank by 50% each day after waste was collected.

### 2.5. Terminal Sampling

Following a four-week exposure, fish were sacrificed by overexposure to thyme oil (100 ppm). Fish length and weight were measured on a measuring board to the nearest 0.1 cm and gram, respectively. Six fish from each replicate were used for collecting data on the length–weight and condition factor parameters. Two fish from each replicate were sacrificed for serum-related analysis and gene expression reactions. Intra-replicate samples were pooled to obtain triplicated treatment samples; hence, a total of three samples per treatment were analyzed. Blood was collected using a syringe from the caudal vein (0.3 to 0.5 mL/fish) for serum Vtg analysis as a potential biomarker of exposure to EDCs. In order to investigate the gene expression (Cyp1A1, Vtg, and 18 s as the housekeeping gene) pattern in the treated fish, different organs (gonad, intestine, gill, liver, and head kidney) were aseptically collected (10–20 mg) and immediately homogenized in an RNA lysis buffer. Protocols of RNA isolation, with a step of genomic DNA denaturation, were followed as described in the kit brochure (Aurum™ Total RNA Mini Kit, BioRad, Watford, UK).

### 2.6. Biological Parameters

The condition factor (CF) was estimated according to [59], and Fulton’s factor was calculated as follows:

CF = (100TW)/(TL^3^)

where CF represents the condition factor, TL represents the total length in centimeters, and TW represents the total weight in grams of the fish.

The hepatosomatic index (HSI) is expressed as the relative liver weight as a percentage of the total body weight [60,61]. The HSI was calculated by weighing livers and dividing them by the total weight of the fish as follows:

HSI = liver weight (g)/total weight (g) × 100

### 2.7. Serum Protein and Vitellogenin

The blood was collected via a caudal vein and stored overnight at 4 °C for serum separation. The blood was centrifuged at 6000 rpm for 2 min to separate the serum.

#### 2.7.1. Serum Protein

Serum was diluted 1:2 in sterile phosphate-buffered saline (PBS pH 7.2) for serum protein estimation using a Nanodrop Spectrophotometer (Thermo Scientific, Waltham, MA, USA) at 280 nm.

#### 2.7.2. Serum Vitellogenin

Serum Vtg was estimated using a dot-immuno assay on nitrocellulose paper (BioRad, Watford, UK). The dot-immuno assay was carried out following methodology from Cardosa and Tio (1991) [62]. Briefly, serum was double-diluted (1:2, 1:4, 1:8, 1:16, etc.) with sterile PBS (pH 7.2) and dotted (5 µL of different dilutions) on nitrocellulose (0.45 µm), then air-dried. Unbound space on the paper was blocked using a blocking solution (2% bovine serum albumin in sterile PBS with 1% tween 20, PBST), then air-dried and reacted with seabream anti-Vtg polyclonal rabbit antibodies (1:5000 dilutions in sterile PBS). The paper was washed 3x using PBST after an incubation time of one hour before being air-dried, and the horse radish peroxidase (HRP)-conjugated secondary antibody (goat anti rabbit 1:5000 diluted) was added to the spotted sites. This underwent reaction for one hour and was washed 3x using PBST. Ready-to-use diaminobenzidine (BioRad, UK) was added after air-drying. Spot development was stopped after 20 min using PBST washing. The spots were photographed, and the titers were determined. The control fish (fed only MPs, unexposed) was used for a comparison of the results and evaluating the effect of treatments.

#### 2.7.3. qPCR Assay for CYP 1A1 and Vtg Genes in Liver Tissue

Total RNA was extracted from homogenized tissue samples using an RNA isolation kit (Aurum total RNA isolation kit, BioRad) with DNAse treatment before elution. The quality and quantity of extracted RNA were evaluated using a Nanodrop analyzer (Thermo Scientific, USA). The extracted RNA was used for synthesizing cDNA using random primers following kit protocols (iScript cDNA kit, BioRad, UK). Primers for the CYP 1A1 [63] and Vtg [64] genes were used for qPCR assays. These primers were previously evaluated using the melt curve analysis for their suitability in yellowfin bream. The qPCR (Mx3005P, Agilent, Santa Clara, CA, USA) assays were carried out using SSo Syber green master mix (BioRad, USA) and an 18 s endogenous reference gene [63] was used as the housekeeping gene. Results were analyzed using the 2^−ΔCT^ method [54]. All reactions were carried out in triplicate.

The primers used in the gene expression were

Vtg A F: 5′AAG TCA AGG CCA CCA CAA CA 3′;

Vtg A R: 5′GCA CAG CTG CAA TGT GTT CA 3′;

Cypi1A F: 5′GCA TCA ACG ACC GCT TC ACGC 3′;

Cyi1A R: 5′CCT ACA ACC TTC TCA TCC GAC ATC TGG 3′;

18 S F: 5′ CTT CAA CGC TCA GGT CAT CAT 3′;

18 R: 5′ AGT TGG CAC CGT TTA TGG TC 3′.

### 2.8. Statistical Analysis

A one-way analysis of variance (ANOVA) was performed to analyse the difference between the means of different treatments with significance at *p* < 0.05.

## 3. Results

The results reported five types of estrogens, namely diethylstilbestrol (DES), estrone (E1), 17-β-estradiol (E2), estriol (E3), and 17-β-ethinylestradiol (EE2), in both WWTP streams (influent and effluent). The total estrogens level was 15.68 ng/g in the influent streams and 4.79 ng/g in the effluent streams (Table 1).

**Figure 2:**
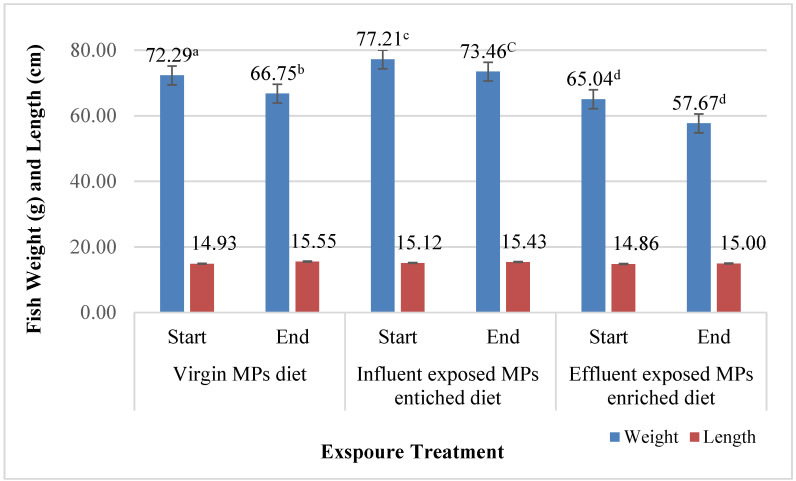
Initial and final total weight and length of dietary exposed fish to LDPE microplastics (bars with common letter are not significantly (*p* > 0.05) different). Error bars represent standard errors.

Prior to dietary exposure, the initial weight and length of the fish in each treatment were calculated (Figure 2).

At the end of dietary exposure, the average total weight and length were recorded for the control group (66.75 ± 9.00 g and 15.55 ± 0.58 cm, respectively) and showed significant decreases in total body weight (*p* = 0.034). However, the total length increased significantly (*p* = 0.0054) after four weeks of dietary exposure. The total length indicated as fish growth based on previous research reported that there is a positive linear relationship between the maximum observed length and the growth rate of several fish in the natural environment [65].

The influent- and effluent-exposed MP-enriched diet groups both followed the same pattern as the control group; however, the final total weight of both groups was non-significantly decreased (Figure 2), although they were given the same mixture of diet (MP-enriched diet). The only difference was that their MPs were exposed to the WWTP (influent and effluent). Both influent and effluent groups showed a non-significant increase in total length (Figure 2). The increase in length was less than in the control group.

**Figure 3:**
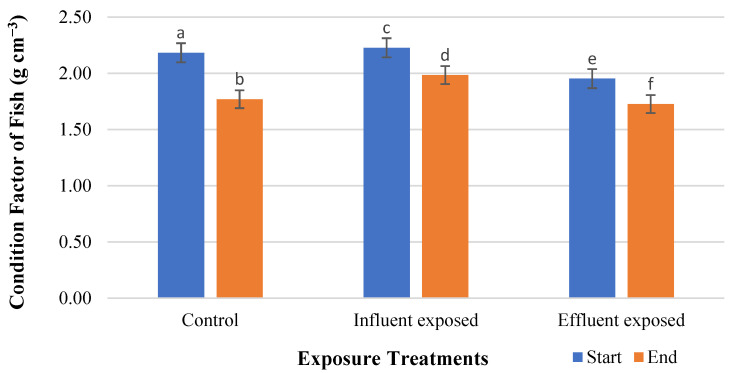
Condition factors of exposed fish from three treatments. a, b, c, d, e, f represent bars with similar bar label are not significantly different from another.

The condition factor (CF) is also known as the length–weight factor. Using Fulton’s factor (Table 2), the condition factor results were calculated for the three treatments (Figure 3). Following the four-week exposure, CF was highly significantly decreased (*p* < 0.05) in all three treatments.

**Figure 4:**
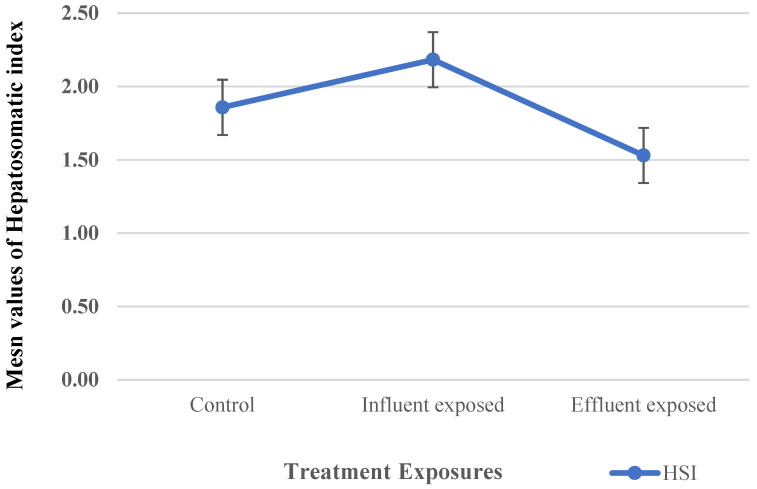
Hepatosomatic index changes by dietary exposure to MPs.

The HSI was investigated for the three treatments as an indicator of environmental stress and exposure to contaminants, which is essential to evaluate the liver damage induced [66] (Figure 4). The results, presented as mean values ± SD, after the terminal sampling showed that the average HSI detected for the control groups was 1.86 ± 0.35. For the dietary treatment group, there was a non-significant increase in the HSI of the influent-exposed MP group (2.18 ± 0.76). In the case of the effluent-exposed groups, the results showed a significant decrease (1.53 ± 0.18) in comparison to the control group.

### 3.1. Biochemical Parameters

#### 3.1.1. Serum Protein and Vitellogenin

**Figure 5:**
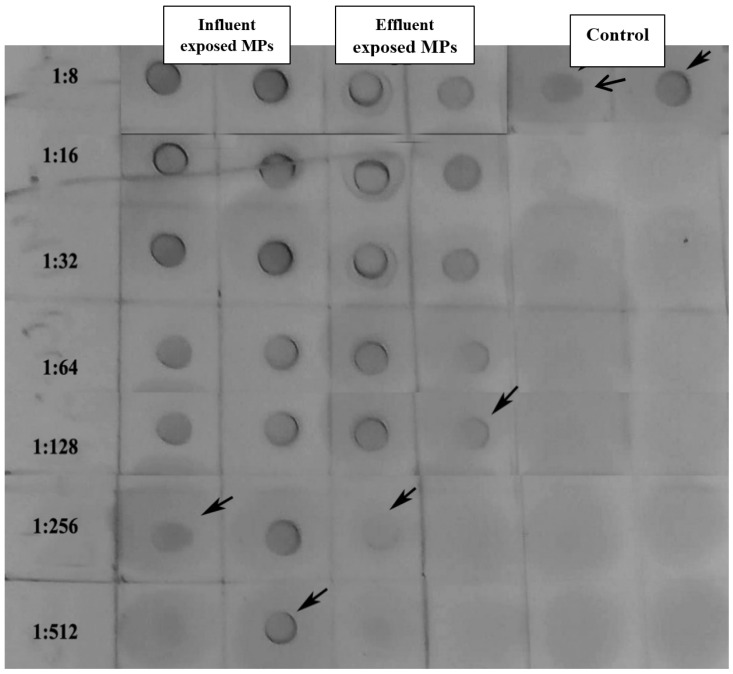
Dot-spot assay of serum Vtg of the three treatment (influent, effluent, and control) dietary groups. (Arrows indicate the highest dilution or titer with a clear precipitin line).

Serum protein levels were not significantly different (*p* > 0.05) in influent and effluent MP-exposed dietary groups compared to the control group. This indicated that the exposure in the control groups did not affect the protein content *per-seI*, but the influence on the vtg content was evident. However, the Vtg dot-spot assay (Figure 5) suggested a difference of at least one log2 dilution between the fish fed MPs exposed to influent water compared with that of effluent water. This indicated a potential carrier effect of MPs that could influence the endocrine functions. The exposed MPs significantly (*p* < 0.05, *t*-test) increased Vtg levels in the serum of fish compared with those of the control group. The difference between the titers of the respective groups was assessed based on the titers showing clear precipitin lines.

#### 3.1.2. qPCR for CYP1A1 and Vtg Genes in Liver Tissue

**Figure 6:**
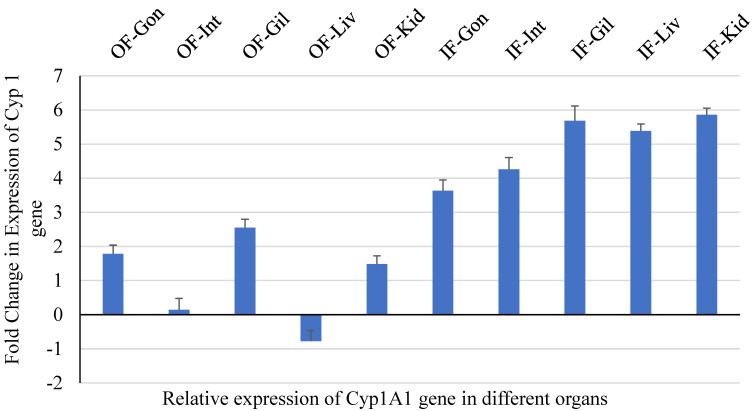
Fold change (relative to control fish) in expression of Cyp 1A1 gene in various tissues (OF—outflow/effluent; IF—inflow/influent).

**Figure 7:**
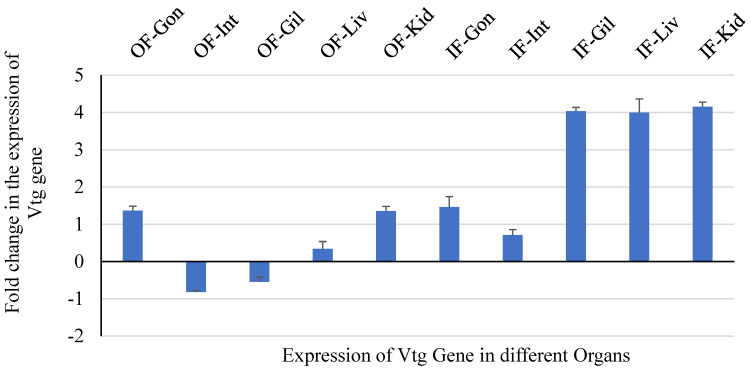
Fold change (relative to control fish) in expression of Vtg gene in various tissues.

Gonad, intestine, gill, heart, liver, and head kidney samples were collected to measure CYP1A and Vtg gene expression following the protocols of Stagg et al. [67]. The expression pattern of the selected genes is depicted in Figure 6 and Figure 7, which were compared with that of the control fish group. Cyp1A gene expression was negatively affected by exposure to influent-exposed MPs, which corresponds to higher levels of MPs in the influent-exposed streams. A healthy liver is known to metabolize organic contaminants with the help of the cytochrome P450 1A (CYP1A) enzyme [68].

## 4. Discussion

Several EDCs are found in municipal wastewater treatment plants, including natural and synthetic estrogens. Most of these hormones are derived from human excretion, including natural hormones such as E1, E2, and E3, as well as synthetic estrogens derived from contraceptives, such as EE2 [69]. The extent to which estrogen is removed during the treatment process depends on the treatment technology [70]. This is due to their chemical stability and incomplete biodegradation [71]. It has been demonstrated that estrogens can be adsorbate on MPs [18]. According to previous studies, virgin LDPE leaks bisphenol A (BPA), whereas older LDPE has a greater ability to bind to BPA in water environments [72]. Kuwait’s most used polymer is LDPE; therefore, it was selected for this study. As a result of its coloration, buoyancy, and resemblance to food, LDPE is particularly attractive to fish [47]. Unlike other types of polyethylene, this type can be readily sourced and does not require special handling. It is reported that polyethylene MPs are common in the stomachs, guts, and livers of commercial fish, and they can cause them physical harm or adverse reactions [26,73,74]. The LDPE MPs were exposed to both the inflow/influent streams and outflow/effluent streams of the Kabd wastewater treatment plant and were subsequently claimed back to the laboratory for use in the preparation of fish feed. As a result of the non-exposed MP-enriched diet, a significant decrease in body weight was observed in the control group. The lack of nutrients provided by the nonedible MPs may have resulted in an effect resulting in an increase in satiety, which affected the total body weight of the control group. Several studies have shown that MPs may block fish digestive tracts, reduce food intake, cause false satiation, decrease caloric intake, and cause a loss of appetite once ingested [75,76].

In terms of the treatment groups, the weight and length followed a similar pattern as the control group. The MPs exposed to WWTPs were found to have estrogen levels as a result of exposure [18,24]. It has been reported that fish growth is regulated by sex steroids and growth hormones. Therefore, the fish can grow throughout life, and body weight slows down considerably during gonadal growth and gamete formation because of energy partitioning [72,77]. A study reported that E2 can promote growth in female fish and reduce muscle growth due to nutrient partitioning during sexual maturation [78]. The estrogen levels detected in the WWTP-exposed MPs could have affected the weight results. A previous study reported that the LDPE pellets showed the highest affinity to E2 [18]. It has been suggested that E2 regulates lipid metabolism in fish muscle.

Also, the liver plays a crucial role in lipid metabolism, which is responsible for somatic growth. Fish with abnormal lipid metabolism suffer from adverse effects on their health, growth, and productivity [77,79,80]. The non-significant increase in length in the treatment groups could be explained by the estrogenic effect that may have been desorbed from MPs. It was reported that fish exposed to sewage effluents grew consistently throughout the experiment, and they had no difference in mean size (length or weight) between treatment groups [81]. Previous studies reported that the ingestion of numerous types of MPs results in weight loss and decreased growth of fish, which is in line with our findings [82,83].

The CF is an organism-level response to variables like nutritional status, pathogen effects, and exposure to toxic chemicals that affect weight differently than normal. The CF measures the degree of the well-being of fish in their habitat. The higher the CF value, the better the condition of the fish. Several factors can affect fish CFs, including stress, season, and food availability [84]. Despite a decrease in the CF during the exposure period, the general CF was still good. The results of our study are consistent with those of another study in which three-spined sticklebacks consumed polyester fibers for nine weeks without affecting their condition factor.

In fish, the HSI is one of the most commonly used indicators of contamination due to the liver’s role as a primary detoxification organ [61]. As a stress biomarker at the organ level, the HSI can be an excellent indicator of adverse health in fish [85]. Pollutants have the potential to affect detoxification organs such as the liver and kidneys [86]. According to a recent study, dietary exposure to two types of polyethylene MPs with different median sizes increased the HSI of farmed tilapia (*Oreochromis niloticus*) [87]. According to this study’s results, the group exposed to effluent MPs had a significant decrease in the HSI. This significant decrease may be interpreted as a reduction in physiological stress as a result of a reduction in exposure to pollutants in effluents following treatment, as estrogen levels detected in MPs fed to this group were lower.

The dot-spot assay revealed a measurable difference in Vtg between the two groups treated with MPs exposed to WWTP. There is a possibility that this is caused by the higher concentration of estrogens extracted from MPs exposed to influential streams as compared to MPs exposed to effluents. The significant difference evidently reflects higher levels of estrogen exposure and inhibition of the CYP 1A enzyme in fish, indicating a reduced ability to detoxify EDCs. A study reported that zebrafish treated with both abamectin (ABM) alone and in combination with MPs showed a significantly decreased gene expression of CYP1A, while the expression of the Vtg gene was significantly increased in samples treated with the combination of ABM and MPs [88].

## 5. Conclusions

Control group body weight decreased significantly as a result of the non-exposed MP-enriched diet. In the control group, the lack of nutrients may have resulted in an increase in satiety, affecting total body weight. A similar pattern was seen in the treatment groups. Estrogen levels detected in WWTP-exposed MPs may have influenced weight results.

Non-significant length increases may be explained by estrogenic effects desorbed from MPs. Despite a decrease in the CF during the exposure period, the general CF remained good. In this study, the group exposed to effluent MPs had a significant reduction in the HSI. As MPs fed to this group showed lower estrogen levels, this may indicate a reduction in physiological stress as a result of reduced exposure to contaminants in effluents. The study showed that LDPE can act as a vector for estrogen and one ingested by fish showed a reduction in control group weight due to the LDPE. In this study, MPs were found to negatively influence yellowfin bream growth; the long-term effects of such exposures will be interesting to observe. Upon the ingestion of MPs, vitellogenin was induced and cytochrome function was negatively affected.

## Figures and Tables

**Table 1:** Estrogens (ng/g) on MPs exposed to effluent streams in Kabd WWTP.

Estrogens	Influent Streams	Effluent Streams
DES	1.13	0.00
E1	8.24	0.96
E2	0.54	0.00
EE2	2.24	3.83
E3	3.54	0.00
Σ Estrogens	15.68	4.79

**Table 2:** Mean values of condition factors of the three exposure treatments; values are expressed as mean ± SEM (significance * *p* < 0.05).

Exposure Time	CF (100 × g cm^−3^)		
	Control	Influent-Exposed	Effluent-Exposed
Start	2.18 ± 0.06 *	2.23 ± 0.05 *	1.95 ± 0.03 *
End	1.77 ± 0.03	1.98 ± 0.03	1.73 ± 0.07

## Data Availability

Data is contained within the article.
